# Utility of Breath-Holding Time in Monitoring Acute Neuromuscular Respiratory Failure in Bulbar-Onset Myasthenia Gravis: A Case Report

**DOI:** 10.7759/cureus.89538

**Published:** 2025-08-07

**Authors:** Saboor Zulfiqar, Aylin Ugurlu, Vicky Moore, Rahul Mukherjee

**Affiliations:** 1 Respiratory Medicine, Birmingham Heartlands Hospital (Part of University Hospitals Birmingham NHS Trust), Birmingham, GBR

**Keywords:** bulbar palsy, myasthenia gravis, physiologic monitoring, respiratory function tests, respiratory insufficiency, vital capacity

## Abstract

We report the management of a 64-year-old male with newly diagnosed bulbar-onset myasthenia gravis (MG) who was hospitalized with acute neuromuscular respiratory insufficiency. This case highlights the challenges in monitoring respiratory function in MG patients, especially in the presence of bulbar and nuchal weakness, and emphasizes the potential utility of single breath-hold time (SBHT) over forced vital capacity (FVC) as a reliable bedside monitoring tool. Despite initial stabilization with intravenous immunoglobulin (IVIG), the patient deteriorated, requiring escalation to the intensive care unit (ICU), and the clinical worsening corresponded with the SBHT rather than with FVC.

## Introduction

Myasthenia gravis (MG) is a chronic autoimmune disorder characterized by fluctuating muscle weakness due to impaired neuromuscular transmission. It affects approximately 14-20 people per 100,000 globally and can present at any age, with a bimodal distribution in incidence [1]. MG is classically divided into ocular and generalized forms, with bulbar-onset MG being a subset that primarily affects the muscles of the face, throat, and neck and presents unique challenges, particularly in monitoring respiratory function. Diagnosis typically involves detection of acetylcholine receptor (AChR) antibodies or muscle-specific kinase (MuSK) antibodies, along with electrophysiological studies such as single-fiber electromyography (EMG). Treatment is aimed at symptom control (e.g., pyridostigmine), immunosuppression (e.g., corticosteroids, azathioprine), and immunomodulatory therapies like intravenous immunoglobulin (IVIG) or plasma exchange during acute exacerbations. During a myasthenic crisis, simple and reliable methods to identify patients at risk of ongoing or worsening respiratory function may enable respiratory support therapies to be offered at an earlier stage to those in need [2]. Respiratory muscle weakness is among the most serious complications of bulbar-onset MG, with up to 15-20% of patients experiencing myasthenic crises early in the disease course, necessitating ventilatory support [3].

During such crises, timely identification of impending respiratory failure is essential to enable early respiratory intervention. Traditional bedside assessments like forced vital capacity (FVC) are widely used, but their reliability can be compromised in patients with bulbar and nuchal weakness due to impaired airway patency and poor mouth seal [4,5]. Moreover, FVC measurement requires calibrated spirometry equipment and staff with specific lung function assessment skills, which may not be available round-the-clock in many medical wards. In contrast, simpler bedside tools such as single breath-hold time (SBHT) and single breath count test (SBCT) have been proposed as alternative monitoring methods [6]. SBHT involves asking the patient to take a maximal inhalation and hold their breath for as long as possible, while SBCT requires the patient to count aloud in a single breath as far as they can in a normal speaking voice. Both tests have shown reasonable correlation with FVC and are easy to perform, even in resource-limited settings or outside of standard working hours [6-8]. However, it is important to note that despite emerging evidence supporting their use, these tools are not yet universally adopted in clinical guidelines. This case report explores the respiratory monitoring challenges in a patient with bulbar-onset MG and illustrates how SBHT served as a more reliable and practical monitoring tool than FVC in predicting clinical deterioration.

## Case presentation

A 64-year-old male with a recent diagnosis of MG presented to the hospital with worsening dysphagia, dysphonia, and increased work of breathing. He lived alone and was fully independent in all activities of daily living (ADLs) before admission. His medical history included obstructive sleep apnea (OSA), keratoconus (left eye), osteoarthritis, and varicose veins. There was no family history of neuromuscular or autoimmune disorders. He lived alone, never smoked, and reported only occasional alcohol consumption. His symptoms had progressed over several weeks, culminating in significant difficulty swallowing solids, frothy green phlegm production, and nasal regurgitation of water. He denied vomiting, chest pain, or other systemic symptoms. The patient also reported severe sleep deprivation due to frequent arousals for clinical observations, exacerbated by a background of OSA. Notably, he had discontinued his continuous positive airway pressure (CPAP) therapy prior to admission. At presentation, although weak, he could transfer and ambulate within his bedspace and perform self-care with effort.

He had a well-documented history of bulbar-onset MG, confirmed by markedly elevated AChR antibody levels (>6 nmol/L; normal range: 0-0.39). Single-fiber EMG had been planned as part of outpatient follow-up, but it was not completed during admission due to clinical instability. Prior to this admission, he had demonstrated a favorable response to pyridostigmine, which provided symptomatic relief.

On admission, the patient was alert and oriented, with a resting oxygen saturation (SpO₂) of 96% on room air. Neurological examination revealed severe nuchal weakness, which the patient described as unchanged from baseline. There was no lateralizing neurological deficit, and he was able to transfer and ambulate independently within the confines of his bed space. Initial bedside respiratory function testing revealed a FVC of 0.22 L, but the patient’s breath-holding time was measured at 30.3 seconds, which was considered safe.

**Figure 1:**
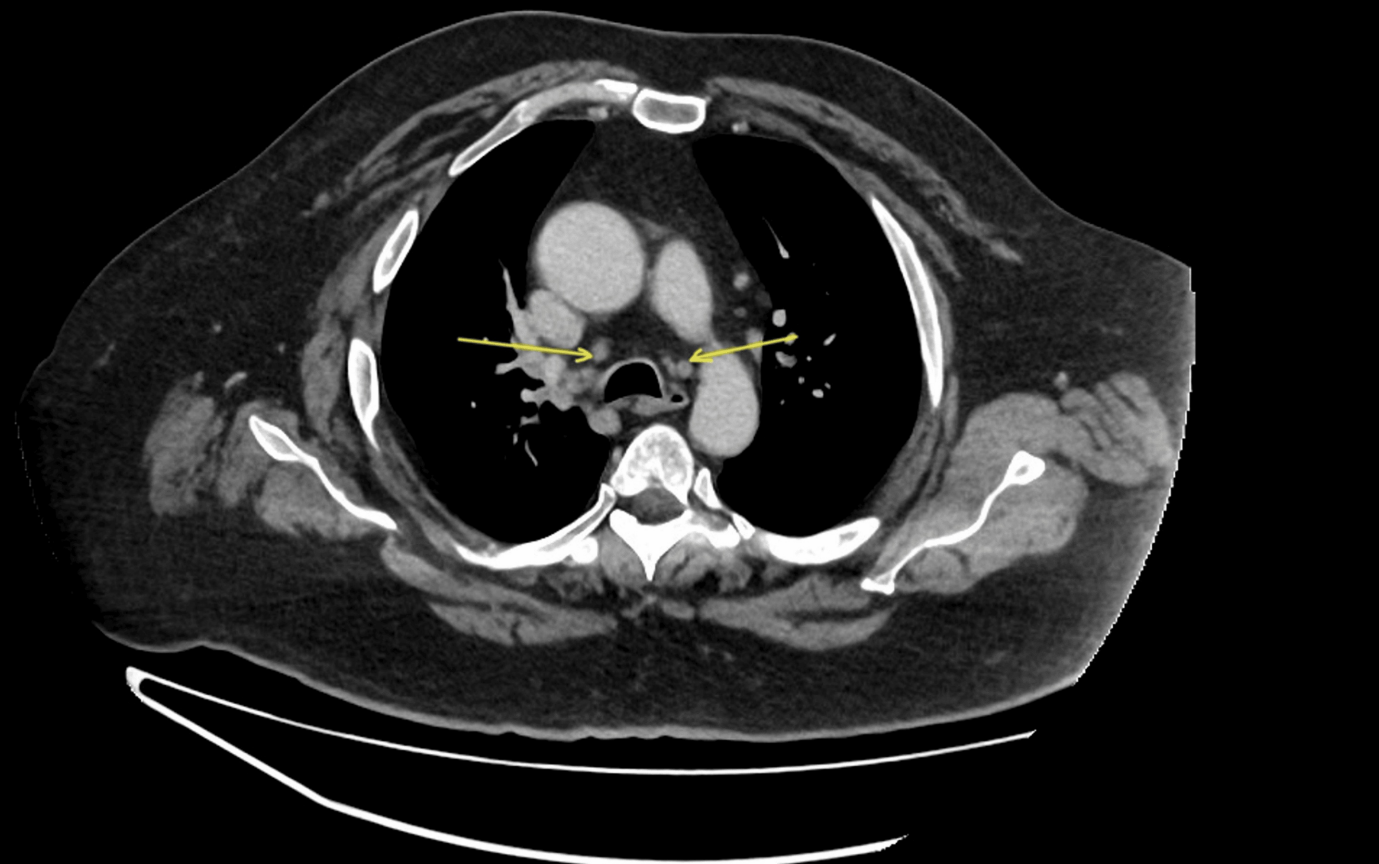
CT thorax showing mediastinal lymph nodes (arrows)

Laboratory investigations revealed no remarkable abnormalities. Full blood count (FBC) and renal and liver function tests were within normal limits, and the C-reactive protein (CRP) level was mildly elevated at 10 mg/L (reference range: <5 mg/L). Chest radiography demonstrated no focal consolidation, and computed tomography (CT) of the thorax (Figure 1) showed small mediastinal lymph nodes and, importantly, ruled out thymoma.

Hospital course

The patient was admitted to the respiratory support ward and commenced on IVIG therapy under the guidance of the neurology team. He was monitored for signs of respiratory deterioration, given the high risk of acute neuromuscular respiratory failure associated with bulbar-onset MG.

Over the next 48 hours, his respiratory function fluctuated significantly, with discordance between FVC and breath-holding time. This divergence prompted clinical concern. Despite initial stabilization, his condition deteriorated, necessitating transfer to intensive care. Key respiratory function test results are summarized in Table 1. Following his transfer to the intensive care unit (ICU) and appropriate escalation of care, the patient made a remarkable recovery. By the time of discharge, he had returned to his baseline functional status, with resolution of respiratory symptoms and restoration of his pre-admission quality of life.

**Table 1:** FVC, SBHT, and clinical state FVC: forced vital capacity; SBHT: single breath-hold time

Date	FVC (L)	SBHT (Seconds)	Clinical notes
Day 1 (AM)	0.22	30.3	Stable breath-holding time. Severe nuchal weakness noted. Patient able to mobilize across bedspace, sit upright, and raise arms above the shoulder
Day 1 (PM)	0.40	15	Significant drop in breath-holding time despite slight improvement in FVC. Patient able to sit upright; unable to raise arms above the shoulder
Day 2 (AM)	1.47	12	Improved FVC, but breath-holding time remained low; unable to sit upright, transferred to critical care

Learning point

The patient’s FVC fluctuated between 0.22 L and 1.47 L during admission, often misleadingly suggesting improvement or deterioration. However, breath-holding time correlated more accurately with clinical status, showing a decline from 30.3 seconds to 12 seconds with worsening upper body weakness when the patient had to be taken to critical care for an impending myasthenic crisis. This case highlights the limitations of FVC in patients with bulbar and nuchal weakness, where airway patency and rectitude are compromised. SBHT, a simple and reproducible bedside test, demonstrated utility as a superior bedside monitoring tool for acute neuromuscular respiratory failure in this patient.

## Discussion

This case underscores the challenges of monitoring respiratory function in patients with bulbar-onset MG in real life, particularly in those with significant nuchal weakness. Traditional respiratory function tests like FVC are often unreliable in this population due to bulbar dysfunction, which may compromise airway patency and interfere with accurate test performance. Inadequate mouth closure or impaired coordination may result in air leaks or inconsistent expiratory effort, potentially leading to artifactually low measurements. Additionally, nuchal weakness can limit the patient’s ability to generate adequate inspiratory and expiratory effort. In our case, we observed that SBHT tracked closely with the patient's observable clinical state and functional capacity, particularly during acute deterioration and subsequent recovery, which aligns with previous studies validating arm-level correlation of SBHT and SBCT with FVC and their utility in monitoring neuromuscular respiratory function [7,9]. We discuss our findings in three broad areas: patient considerations, correlation with clinical state, and implications for practice.

Practical considerations

**Figure 2:**
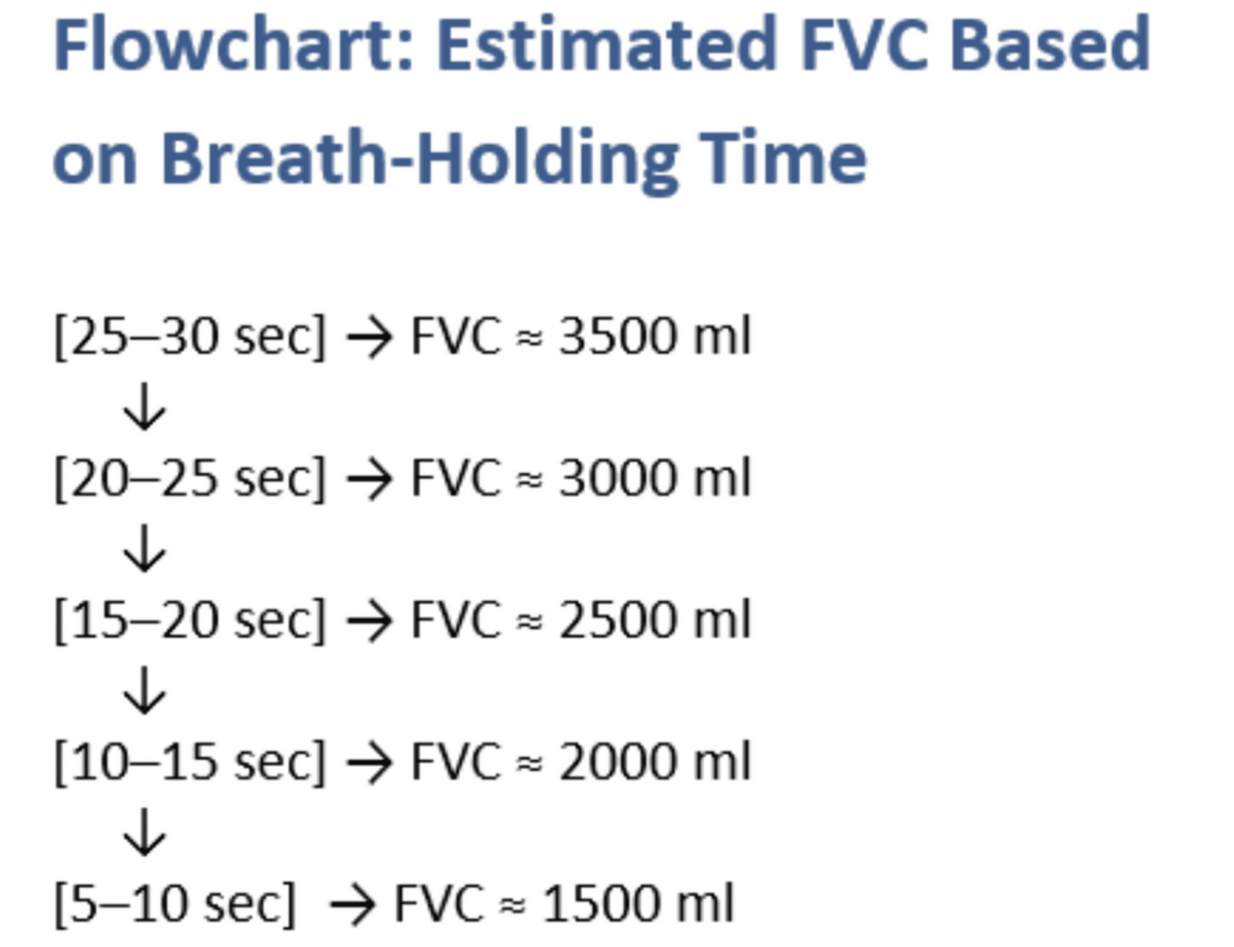
Estimated forced vital capacity (FVC) based on single breath-hold time Image credit: Saboor Zulfiqar. Image created by the author based on data trends described in Snider TH [6].

SBHT (Figure 2) is a reliable and practical bedside tool for assessing respiratory muscle strength and predicting impending respiratory failure [6,10]. It is simple to perform and requires no specialized equipment, making it particularly useful during out-of-hours monitoring when respiratory physiology expertise and calibrated spirometry devices may be unavailable. Our internal audit of 10 patients requiring respiratory function testing over seven months demonstrated a 67% correlation between SBHT and FVC values, calculated using Pearson’s correlation coefficient based on paired data points. However, the small sample size and limited data points mean this correlation should be interpreted cautiously and requires validation in larger studies. The audit also highlighted challenges in maintaining regular equipment calibration and respiratory physiology skills on medical wards outside working hours, reinforcing the need for simple, reliable bedside measures like SBHT.

Correlation with clinical state

In this case, the patient’s FVC fluctuated between 0.22 L and 1.47 L during admission, which at times misleadingly suggested improvement or deterioration. In contrast, SBHT showed a decline from 30.3 seconds to 12 seconds, correlating closely with the patient’s worsening clinical status and need for critical care transfer. This divergence highlights the limitations of FVC in bulbar and nuchal weakness, where airway patency and patient effort may be compromised. The moderate correlation between SBHT and FVC values observed in our audit and case is consistent with earlier literature, but further robust evidence is required to define the precise role of SBHT in clinical monitoring.

Implications for practice

This case supports the use of SBHT as a complementary monitoring tool in patients with neuromuscular respiratory insufficiency, particularly where FVC is unreliable or difficult to obtain. The SBCT, which has been proposed as an alternative bedside measure with good correlation to FVC and sensitivity in predicting respiratory decline [9,11], was mentioned but not systematically applied in this case; hence, our focus remains on SBHT. Limitations of SBHT include its reliance on patient cooperation, potential variability across observers, and the impact of cognitive or language barriers. These should be considered when interpreting results in clinical practice [10,11]. Treatment decisions and follow-up in this case were guided by clinical deterioration aligned with SBHT changes, with immunotherapy escalation and transfer to critical care. Future studies should explore how bedside respiratory measures like SBHT can be integrated into standardized monitoring and treatment algorithms.

While much of the foundational literature on SBHT is dated [6], newer studies continue to support its utility in resource-limited or out-of-hours settings [7-9]. Our findings contribute to this growing evidence base and highlight the need for further prospective research to validate SBHT as a reliable marker of respiratory muscle function in bulbar-onset MG.

## Conclusions

Our case highlights the importance of reproducible and reliable monitoring strategies in patients with MG and acute neuromuscular respiratory failure. For this patient, SBHT proved to be a useful indicator of respiratory function, correlating more closely with clinical status than FVC, guiding timely escalation of care to the ICU. While SBHT shows promise as a practical bedside tool, it should be considered complementary to, rather than a replacement for, standard respiratory function tests. Given that this report is based on a single case and a small internal audit, further prospective studies are needed to validate the utility of SBHT in wider clinical practice. We recommend that SBHT measurements be recorded alongside FVC to provide more comprehensive monitoring, especially in settings where access to formal respiratory physiology is limited. Disseminating awareness of simple, reliable bedside measures like SBHT may support safer, more timely care in patients with acute neuromuscular respiratory insufficiency, particularly in resource-limited settings.
